# Successful Treatment With High-Dose Colchicine of a 101-Year-Old Patient Diagnosed With COVID-19 After an Emergency Cholecystectomy

**DOI:** 10.7759/cureus.63201

**Published:** 2024-06-26

**Authors:** Dimitar Bulanov, Atanas Yonkov, Elena Arabadzhieva, Vanyo Mitev

**Affiliations:** 1 Department of General and Operative Surgery, Medical Faculty, Medical University - Sofia, Sofia, BGR; 2 Department of Medical Chemistry and Biochemistry, Medical Faculty, Medical University - Sofia, Sofia, BGR

**Keywords:** general surgery, age, colchicine, cytokine storm, nlrp3 inflammasome, covid-19

## Abstract

There are multiple factors associated with increased morbidity and mortality in COVID-19 patients, and advanced age is one such independent prognostic factor. It is well established that the multiorgan failure and death in COVID-19 patients are due to the hyperactivation of the NOD-, LRR- and pyrin domain-containing protein 3 (NLRP3) inflammasome and the ensuing cytokine storm. Colchicine, a well-known anti-inflammatory drug, has been shown to inhibit the NLRP3 inflammasome in micromolar concentrations potently. It has the unique property of accumulating in leukocytes, which is the primary cause of the abnormal activation of the NLRP3 inflammasome in COVID-19. It has been shown that achieving inhibitory concentrations of colchicine in leucocytes requires treatment with higher doses. Our recent studies showed that treatment with higher doses of colchicine in both outpatient and inpatient settings is safe and results in remarkable cure rates and significantly decreased mortality rates, even in the most severely affected patients with multiple comorbidities and risk factors.

The main risk factor for severe COVID-19 is age, especially over 85 years. Here, we present a unique case of a 101-year-old male who underwent two major emergency abdominal surgeries and contracted COVID-19 while in the hospital. Laboratory tests showed increased values of markers for severe COVID-19, including CRP, D-dimer, and ferritin. Increased opacities bilaterally paracardially and moderate right-side pleural effusions were detected on the chest X-ray. We initiated our high-dose colchicine treatment regimen, resulting in the patient's complete recovery and discharge. We are convinced that the administration of high-dose colchicine to high-risk COVID-19 patients should be mandatory.

## Introduction

Several studies have pointed out the particular vulnerability of older adults, with a higher proportion of severe cases of COVID-19 and fatal outcomes [1-3]. Advanced age is an independent negative prognostic factor for COVID-19 mortality [1]. The mortality rate observed in patients aged ≥ 85 years of age exceeded 2.8-fold the rate for adults aged 75-84 and sevenfold the rate for adults aged 65-74 [2]. Thus, octo- and nonagenarians, in whom comorbidities such as cardiovascular atherosclerosis, hypertension, chronic lung diseases, and diabetes are highly prevalent, have been associated with the highest proportion of severe COVID-19 cases with fatal outcomes [3]. COVID-19 death rates were higher for men than women of all races among adults aged 65 and over [2]. We have previously demonstrated that using high-dose colchicine for severely ill COVID-19 inpatients leads to a decreased mortality rate between two and sevenfold, depending on the dosage [4-7]. The explanation for the effect of high doses of colchicine is that only in this way can the NOD-, LRR- and pyrin domain-containing protein 3 (NLRP3) inflammasome, whose hyperactivation is responsible for the cytokine storm and subsequent multiorgan damage and death, be inhibited [8].

Here, we present a unique rescue case of a 101-year-old patient who, after undergoing two major surgeries, contracted COVID-19 in the hospital and was discharged healthy thanks to treatment with high-dose colchicine.

## Case presentation

A 101-year-old male patient (weight 70 kg) was admitted with worsening right upper quadrant (RUQ) abdominal pain, nausea, vomiting, and fever for the last four to five days. The patient’s history was significant for hypertension, kidney stones, and chronic gastritis.

Upon admission, he was septic, in acute distress, and hypotensive, with blood pressure in the 70s/40s and a heart rate of 110 per minute. The abdomen exam revealed severe palpatory tenderness in the RUQ with a positive Murphy’s sign. Routine laboratory tests revealed significantly elevated inflammatory markers (increased WBC, C-reactive protein (CRP), fibrinogen, and D-dimer) and slightly increased creatinine and direct bilirubin. Arterial blood gas analysis showed metabolic alkalosis. (Table 1) The O2 saturation was 94% in room air. The result from the SARS-CoV-2 oropharyngeal swab test was negative. The ultrasound of the abdomen revealed an enlarged gallbladder without stones, only with intraluminal sludge and a thickened double-layered wall, grade III according to Tokyo guidelines. The pancreas and the intra- and extrahepatic biliary trees were unremarkable.

**Table 1:** Findings in the patient’s blood test at the time of hospital admission INR: international normalised ratio; pH: potential hydrogen; pCO2: partial pressure of carbon dioxide; pO2: partial pressure of oxygen; SatO2: saturated oxygen

Blood test	Patient’s blood test results	Reference range
Haemoglobin	174	135-180 g/L
WBC	28.1×10^9^	3.5-10.5x10^9 ^/L
Platelets	376×10^9^	150-400x10^9 ^/L
INR	1.1	0.9-1.2
C-reactive protein (CRP)	253.5	<5 mg/L
Fibrinogen	7.9	2-4 g/L
D-dimer	2.11	0-0.55mg/L
Total bilirubin	18	0-21 μmol/L
Direct bilirubin	12.7	0-6.5 μmol/L
Creatinine	144	62-106 mmol/L
pH	7.52	7.35-7.45
SB	25.7	21-25 mmol/L
SatO2	94%	94-98 %
pCO2	3.36	4.67-6 kPa
pO2	8.07	10-13 kPa

Intravenous fluid resuscitation, proton pump inhibitor (PPI) prophylaxis, and intravenous broad-spectrum antibiotics (metronidazole 3x500 mg daily and meropenem 3x1 g daily) were initiated. His general condition and vital signs improved; however, the abdominal status worsened with positive rebound tenderness. A decision was made to proceed with cholecystectomy. Intraoperatively, acute gangrenous cholecystitis with micro-perforations and local subhepatic effusion was found. A cholecystectomy with drainage of the abdominal cavity was performed. Seven days later, the patient underwent a re-operation due to a bile leak. A leak from a small bile duct in the liver bed of the gallbladder (Luschka’s duct) was found and sutured, with subsequent placement of biliary drainage, lavage, and drainage of the abdominal cavity. The patient’s condition and mobility gradually improved. Physical therapy and inpatient rehab slowly progressed to completing the sit-to-stand position.

**Figure 1:**
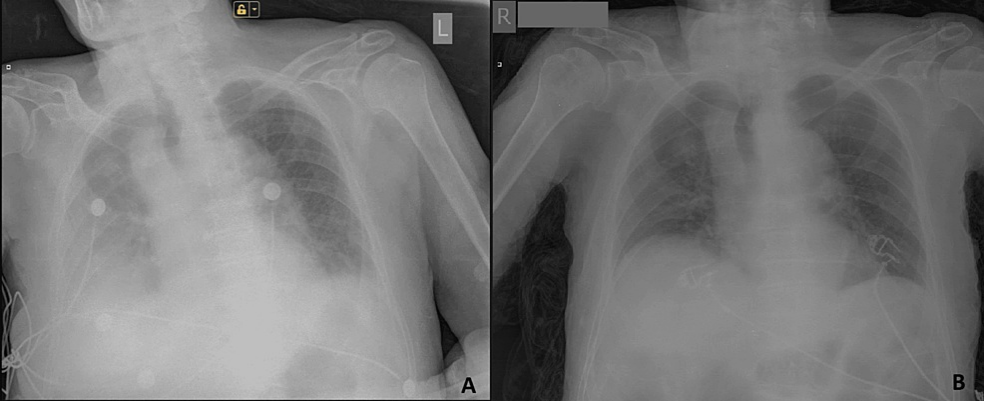
Chest X-ray A: on the day of the COVID-19 diagnosis; B: on the 14th day after treatment with high-dose colchicine was started.

However, 17 days after his re-operation, he started experiencing a cough with mild dyspnea and a fever of up to 38^O^C. A repeated SARS-CoV-2 oropharyngeal swab test confirmed that the patient had COVID-19. The chest X-ray at that time revealed increased opacities bilaterally paracardially and moderate right-side pleural effusion (Figure 1A). Laboratory tests showed an absolute lymphocyte value of 0.6 x 109/L, suggesting the patient’s decreased immunity (Table 2). After a consultation with a pulmonologist, colchicine therapy was initiated (eight tablets (0.5 mg per tablet) daily for two days, then six tablets on day three, five tablets on day four, four tablets on day five and day six, and three tablets daily until day 28 of the COVID-19 diagnosis). Antibiotic treatment with cefoperazone/sulbactam 1 g twice daily was started, and anticoagulant prophylaxis with enoxaparin 4000 IU (40 mg)/0.4 ml, intravenous maintenance fluids, and PPI were continued. He required 3-4 L/min of oxygen via nasal cannula, with SpO2 ranging from 93% to 95%. The patient’s electrocardiogram (ECG), SpO2, heart rate, and arterial blood pressure were continuously monitored. Two days after starting the colchicine therapy, the patient became afebrile. On day six, after the COVID-19 diagnosis, an atrial fibrillation with a tendency to hypotension was registered on the patient’s monitor. Oxygen requirements via nasal cannula gradually improved to 2 L/min with an excellent SpO2 of 99%. After a consultation with a cardiologist, antiarrhythmic and inotropic therapy were initiated. Two days later, the cardiac sinus rhythm was restored, the arterial blood pressure was stable, and SpO2 was 94% on room air. There was an asymptomatic ST depression on the ECG with increased troponins and D-dimer that required heparin drip and nitrates. Within the next three days, the patient improved significantly with a blood pressure of 120/70, a pulse rate of 80 beats per minute, and a SpO2 94-95% on room air without dyspnea. The troponin values were decreasing (Table 2). The anticoagulant therapy was switched back to enoxaparin 6000 IU (60 mg)/0.6 ml twice daily. During the next few days, the levels of troponin and D-dimer decreased. The dynamics of the findings in the blood tests are shown in Table 2.

**Table 2:** Dynamics in findings in the blood tests at the time of COVID-19 diagnosis and afterwards CK-MB: creatine kinase myocardial band

	Day of diagnosis of COVID-19	8^th^ day after diagnosis of COVID-19	14^th^ day after diagnosis of COVID-19	Reference range
Haemoglobin	102	109	101	135-180 g/L
C-reactive protein (CRP)	73	54.1	38.4	<5 mg/L
D-dimer	7.49	8.39	5.51	0-0.55mg/L
Fibrinogen	5.2	4.1	2.9	2-4 g/L
Procalcitonin	0.152	0.097	0.054	0-0.046 ng/ml
Ferritin	680	427	350	13-150 μg/L
Troponin	0.087	0.105	0.051	0-0.014 ng/ml
Leucocytes (WBC)	4.6	9	8.5	3.5-10.5x10^9 ^/L
Lymphocytes	0.6	0.3	0.8	1-4x10^9^/L
Creatine kinase (CK)	59	59	26	0-190 U/L
CK-MB	20	74	22	0-25 U/L
Creatinine	137	123	107	62-106 mmol/L

The follow-up chest X-ray showed a significant improvement (Figure 1B). On day seventeen, after the COVID-19 diagnosis, the patient was discharged without complaints. The therapy with colchicine was continued for 28 days. One month later, the patient was doing well, without any signs of dyspnea, and the biliary drainage was removed.

## Discussion

Our team has been struggling for years to prove that high doses of colchicine are life-saving in severe COVID-19 [4-10]. Our therapeutic strategy is based on inhibiting SARS-CoV-2 entry into the cell (by bromhexine hydrochloride) and inhibiting the NLRP3 inflammasome (by colchicine), two readily available, cheap, and well-known medications. Bromhexine is most effective when given prophylactically or started by inhalation after contact with a person with COVID-19 [8]. However, when the inflammasome is hyperactivated, only high doses of colchicine can prevent or interrupt the cytokine storm.

Several studies, including retrospective, observational, non-controlled/non-randomized, and randomized/controlled trials, have tested the role of colchicine for COVID-19 patients with rather inconsistent and conflicting results [8,11,12]. However, it is essential to underscore that all of these studies tested only low doses of the drug (up to 2.5 mg), most likely because these are the doses usually prescribed to patients with gout flair. The negative results of the largest of these clinical trials, called RECOVERY, led to the ”strong recommendation against“ the use of colchicine for the treatment of COVID-19 issued by the WHO [11,13]. It remains unclear why only low doses of colchicine were used in these studies [14]. Another logical question that begs a somewhat rhetorical answer is why all these research groups have been setting goals to possibly achieve different results while using the same low doses of colchicine and why none have ever tested higher allowable doses of the drug in COVID-19 [8].

A well-established fact is that the cause of COVID-19 complications is the hyperactivation of the NLRP3 inflammasome in leukocytes [15], which is inhibited by high concentrations of colchicine. These concentrations are achievable in white blood cells, where colchicine preferentially accumulates [8].

Another crucial question is: Are the high colchicine doses dangerous? Our maximum loading dose is 5 mg, or 0.045 mg/kg body weight [4,16]. Doses below 0.1 mg/kg body weight are entirely safe, and those below 0.2 mg/kg can, in rare cases, be toxic but not fatal [16,17].

We have published a series of clinical cases demonstrating the life-saving effect of colchicine: a patient in whom only about 10% of the lung was not affected by severe bilateral pneumonia and acute respiratory distress syndrome recovered after treatment with high-dose colchicine [4]; obesity (body mass index (BMI) over 40 kg/m2) is an independent risk factor and is among the significant factors in endothelial dysfunction, the critical pathophysiologic event leading to increased mortality in COVID-19 [9,10,18]. An inpatient (BMI 46.8 kg/m) with type 2 diabetes mellitus and hypertension deteriorated during the standard therapy. When colchicine was included at a dose of 6 mg, the inpatient quickly recovered [9]. Three high-risk patients with a BMI over 50 and 60 kg/m also responded dramatically to 5 mg of colchicine [10]. It is interesting to note that accidentally taking a single overdose of colchicine (15 mg or 12.5 mg) is sufficient for the complete recovery of patients, including the eradication of pericardial effusion [6]. This case shows that in critical situations, higher doses of colchicine can be used [9] but lower than 0.1 mg/kg [16,17].

Limited knowledge exists about the incidence of COVID-19 in patients following surgery. Moreover, there is even less understanding regarding the risk factors and complications associated with COVID-19 in the postoperative phase; however, many factors may have a role [19]. First, the SARS-CoV-2 tests have potential false negative outcomes in a small percentage of cases. Second, older individuals may experience a more extended incubation period with a slower progression to a detectable viral load. Third, individuals can contract COVID-19 during the postoperative period [19]. In our case, the patient was diagnosed with COVID-19 on the 24th day of admission, indicating that this was a nosocomial infection.

Our case highlights the potentially life-saving effect of timely initiated therapy with appropriate high-dose colchicine in severely deconditioned surgical patients with multiple comorbidities and advanced age. In our recent study of 785 inpatients, we have shown that higher doses of colchicine are dose-dependently associated with a two- to seven-fold decrease in mortality [5,7].

All these recently published data support our thesis about the life-saving effect of colchicine not only in COVID-19 but also in other viral infections, such as seasonal influenza-associated with hyperactivation of the NLRP3 inflammasome and its subsequent cytokine storm [10,20].

## Conclusions

This article outlines the principles of what we believe is the only successful treatment for COVID-19. A cause of the complications of COVID-19 is the cytokine storm generated by the abnormally activated NLRP3 inflammasome. In our opinion, the strategy of looking only for antiviral agents is ineffective because there is no direct relationship between viral load and hyperactivation of the NLRP3 inflammasome. None of the WHO-recommended medications for severe COVID-19 can compare effectiveness to high-dose colchicine. High colchicine doses should be strongly considered in the therapeutic regimen of diseases associated with hyperactivation of the NLRP3 inflammasome to prevent cytokine storms, multiorgan failure, and death.
